# Studying the Role of Vegetarianism as a Potential Strategy for Cancer Prevention and Treatment, a Bibliometric Analysis

**DOI:** 10.3390/epidemiologia6020023

**Published:** 2025-05-05

**Authors:** Maria Chrysafi, Maria Gialeli, Constantinos Giaginis, Andreas Y. Troumbis, Georgios K. Vasios

**Affiliations:** 1 Department of Food Science and Nutrition, University of the Aegean, 81400 Myrina, Lemnos, Greece; m.chrisafi3@gmail.com (M.C.); vasios@aegean.gr (G.K.V.); 2 Department of Environment, University of the Aegean, 81100 Mytilene, Lesbos, Greece; atro@aegean.gr

**Keywords:** vegetarianism, vegetarian diet, cancer, neoplasms, bibliometric analysis, Bibliometrix

## Abstract

Vegetarianism, as a dietary pattern, is characterized by animal product avoidance and increased consumption of fruits, vegetables, whole grains, and legumes. It has been associated with health benefits, both physical and psychological, and has raised interest as a potential strategy for cancer prevention and treatment, which remains one of the leading causes of morbidity and mortality worldwide, demanding continual exploration of novel approaches. Background/Objectives: This study aims to describe trends in scientific publications about the relationship between vegetarianism and cancer and to highlight research gaps using bibliometric analysis. Methods: The methodology includes comprehensive research of three literature databases. After combining and cleaning these data, a final sample of 3427 studies was obtained that was analyzed using the Bibliometrix-R package. Results: The results indicate a continuously growing production of scientific publications. The most impactful sources, authors and their collaborations were identified. Author keywords, their co-occurrence network, and thematic trends were studied. Conclusions: Through synthesizing and critically evaluating insights from the scientific literature, we aim to contribute to the understanding of the potential benefits of vegetarianism in cancer prevention and management. However, due to the complexity of the topic, the results are often contradictory and could be used as a starting point for further research.

## 1. Introduction

### 1.1. Rationale

#### 1.1.1. Vegetarian Diet

Vegetarianism is a dietary pattern characterized by the avoidance of meat, poultry, fish, and their products, with variations based on the consumption or exclusion of dairy, eggs, and honey. The four main types of vegetarianism are:

1. Lacto-ovo-vegetarian diet: Excludes meat, poultry, fish, and seafood but includes eggs and dairy products.
2. Lacto-vegetarian diet: Includes dairy products but excludes eggs, meat, poultry, fish, and seafood.
3. Ovo-vegetarian diet: Excludes meat, fish, seafood, and dairy but includes eggs.
4. Vegan diet: Strictly avoids all animal-derived foods, including meat, eggs, dairy, and honey. Subtypes include raw vegan and whole-food vegan diets [1,2].

The adoption of a vegetarian diet has gained popularity in recent years for various reasons, including health, ethics, environmental protection, sustainability, religion, and animal welfare [3,4,5,6]. While vegetarians constitute less than 10% of the population in most countries, exceptions exist, such as India, where approximately 20–39% of adults follow a vegetarian diet primarily for religious and cultural reasons [3,4,6].

Vegetarianism has been associated with significant health benefits, including reduced mortality, a lower incidence of type 2 diabetes, and a lower risk of cancer. Additionally, this dietary pattern appears to positively impact cardiovascular diseases, blood pressure, lipid levels, metabolic syndrome, overweight, obesity, and oxidative stress. It has also been correlated with improved emotional and physical well-being, reduced depression, enhanced quality of life, and overall better health. Furthermore, a plant-based diet significantly influences the composition and function of the gut microbiota, impacting various aspects of life and health [3,7,8,9,10,11,12,13,14]. The health benefits of vegetarianism are largely attributed to its composition, as plant-based foods are rich in phytochemicals with chemoprotective properties [3,15].

#### 1.1.2. Cancer

Cancer represents a complex and multifactorial disease, standing as the predominant cause of mortality on a global scale. In the year 2018, the afflicted population exceeded 12.1 million individuals [16]. By 2020, cancer accounted for an approximate total of 10 million deaths worldwide, with lung, liver, and stomach cancers being prevalent causes of fatality among males and breast, lung, and cervical cancers manifesting as the respective prominent forms in females [4]. Concurrently, it was estimated that around 50.5 million people were living with cancer diagnosed within the preceding five-year period [17].

The genesis of cancer is subject to a confluence of genetic and environmental determinants, with diet emerging as a pivotal, if not paramount, modifiable risk factor [18]. Additionally, adopting a healthy dietary pattern based on plant-derived foods is considered one of the essential guidelines for individuals with or surviving cancer [16,19]. It is estimated that 30–50% of cancer incidences could have been averted through appropriate prevention strategies and avoidance of risk factors such as smoking, alcohol consumption, overweight and obesity, exposure to radiation, and an unhealthy dietary regimen [4].

Devotees of a vegetarian lifestyle are predisposed to adhere to a healthy lifestyle, maintaining body weight, engaging in regular physical activity, and avoiding smoking and excessive alcohol consumption compared with those consuming animal products. This profile is inversely associated with cancer incidence [1,5,20]. An increased intake of nuts, fruits, vegetables, and whole grains is associated with a reduced risk of cancer incidence and mortality. Conversely, consuming small amounts of red meat daily significantly increases the risk of various [5] cancers, whereas the daily consumption of processed meat further increases the risk of cancer-related mortality [3].

Avoiding the consumption of meat and processed meat coupled with an increased intake of bioactive compounds with protective and antioxidant properties, such as phytochemicals, dietary fibers, sulfur compounds, and vitamins, constitutes a potential regulatory axis of critical cancer-related mechanisms, including inflammation, oxidative stress, and cell proliferation [4,16,18,21,22]. On the contrary, due to avoiding specific food groups, this dietary pattern is characterized by a reduced intake of pivotal nutrients, mainly iron, essential fatty acids, and vitamin B12. In certain studies, the deficiency of these dietary components has been implicated in a higher incidence of cancer [22].

The results of studies on the impact of vegetarianism on cancer are contradictory. Some studies, such as the EPIC-Oxford study, suggest a protective effect of vegetarianism against cancer, while others find no correlation or even an adverse impact for certain cancer types [19,23,24,25,26,27,28]. Clarity emerges when considering the quality of the diet. A healthy vegetarian dietary pattern reduces cancer mortality, while an unhealthy vegetarian diet has the opposite effect [17,19,22].

The prevailing uncertainty and confusion within the scientific literature addressing the impact of vegetarianism on cancer underscore the need for further investigation [16]. The increasing number of scientific publications makes accessing and staying informed about the most recent scientific knowledge challenging. This emphasizes the growing importance of literature reviews in synthesizing research findings, promoting a research direction, and providing evidence-based recommendations. Considering the growing interest in the potential anticancer properties of vegetarianism, this study initiates a comprehensive bibliometric investigation. Therefore, bibliometric analysis is of great importance for the health sciences and could complement systematic reviews because it actually identifies the research trends, the emerging topics, and the areas of growing interest while at the same time evaluating the impact of research and map the collaborations and networks, providing useful detailed quantitative information to understand the field and provide guidance for future directions.

### 1.2. Objectives

The present study aims to examine and present the most contemporary scientific approaches regarding vegetarianism in cancer through bibliometric analysis. To achieve this, a comprehensive bibliometric analysis of the scientific literature related to this specific topic will be conducted. This research has a dual objective: first, to critically gather and evaluate the rich scientific material generated by studies exploring the complex relationship between vegetarian diets and cancer, and second, to provide valuable insights into the trajectory of research activities within this field and identify potential prospects for future research.

## 2. Materials and Methods

### 2.1. Bibliometric Analysis

Bibliometry is a metric field practiced for over a century to study the literature’s growth. Even if the first bibliometric studies were performed in the 1910s, it was later defined in several ways [29]. The French term ‘bibliométrie’, by Paul Otlet, was later followed by the definition of Pritchard as “the application of mathematics and statistical methods to shed light on the processes of written communication and on the nature and course of development of a discipline” [30,31].

As the volume of scientific publications continues to grow exponentially, bibliometric analyses offer a systematic means of extracting supporting data for research trajectories and informing strategic decision-making within the scientific community. For these reasons, the use of bibliometry is gradually being extended to all sciences. The mapping of science is complex and difficult because it requires many steps and many different analyses and software tools, not all of which are necessarily freely available [32,33].

To implement science mapping with bibliometric methods, a five-step procedure for conducting science mapping is proposed. First, researchers should define the research questions and choose the appropriate bibliometric methods that can answer them. Second, researchers need to select a database that contains bibliometric data. Third, bibliometric software is employed for analysis. The results of the bibliometric analysis can be further analyzed with statistical software to identify document subgroups that represent research specialties. Fourthly, researchers must decide which visualization method is to be used on the results of the third step and employ appropriate software to prepare the visualization. Finally, the results must be interpreted and described [34].

### 2.2. Information Sources

Considering the above, after determining the purpose of this study and developing the research questions, we began data collection. Data were collected from three different databases containing the scientific literature: PubMed (National Library of Medicine, United States), Scopus (Elsevier, The Netherlands), and Web of Science (WoS). One of the first and most basic steps was to investigate and define the appropriate keywords and the combinations of these to be used so that the results of the searches in the databases would show all the results that are relevant to the topic, not to omit any relevant research and to reduce the possibility of duplicate results. For the analysis, the Bibliometrix R package was used to analyze and visualize the below-interpreted data [33].

The characteristics of the various literature databases, including coverage, focus, and features, may vary from database to database. While Scopus and Web of Science are multidisciplinary, PubMed is primarily focused on biomedical and life sciences fields [35]. In comparison with other bibliometric databases, WoS, Scopus, and PubMed databases provide more information (such as funding) for the indexed articles. Another difference between the databases is the requirement of subscription. Whereas Scopus and WoS require a subscription, PubMed is a publicly accessible database. Out of the three mentioned databases, Scopus indexes the largest number of publications [36]; meanwhile, PubMed is the most reliable when it comes to labeling the types of documents [37]. It is worth mentioning that, until 2004, when Scopus was released, the WoS database was the main source of bibliometric data since 1997 [38].

Therefore, the above-mentioned databases were chosen to identify and collect the relevant scientific articles to find and include as large a range of scientific data as possible. The different approaches of these databases, such as PubMed being focused on health sciences, WoS offers more detailed citation analysis and greater coverage in years, while Scopus includes a wider range of scientific sources than either of the two databases mentioned above, offering diversity in the studies included. The alternative of Google Scholar was rejected as it very often contains insufficient and incorrect information and is not updated systematically [39,40]

### 2.3. Software

In this paper, the bibliometric analysis was carried out using the Bibliometrix R package version 4.0. As an open-source scientometric software tool, it provides a variety of statistical and graphical techniques. Moreover, it is flexible and can be integrated with other statistical R packages. It is, therefore, useful in an ever-changing field such as bibliometrics and was characterized as a standout. Moreover, the Biblioshiny application was used to import the final dataset and to analyze and convert it into understandable charts. The Biblioshiny application provides a web-based user-friendly interface for Bibliometrix. These features were the main reasons for choosing Bibliometrix for the bibliometric analysis [33,41].

### 2.4. A Protocol for Scoping Review

**Figure 1:**
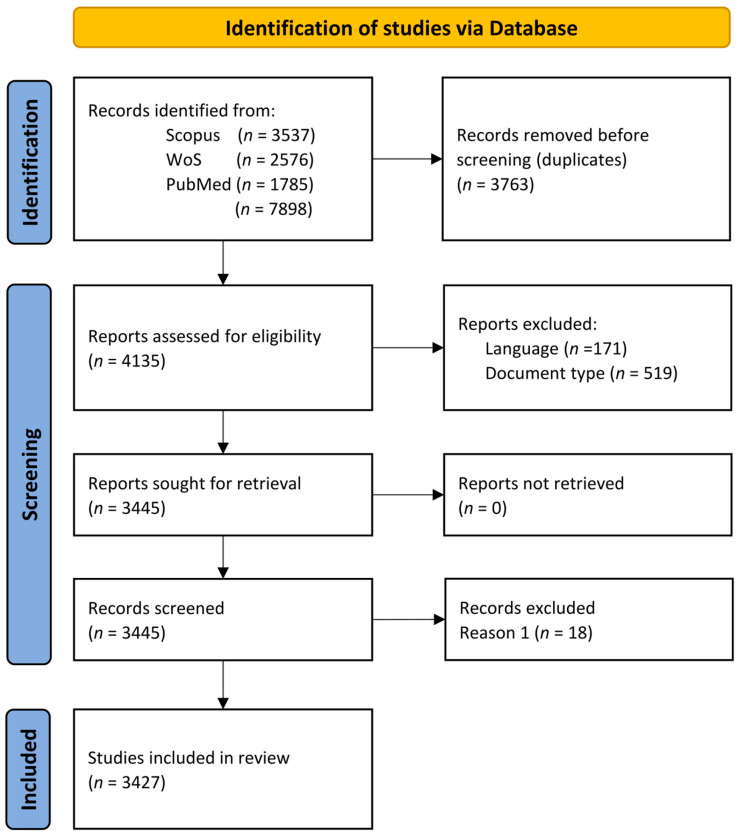
Flowchart indicating the identification and selection of studies used in the present analysis.

The protocol of this review was drafted based on the guidelines of the PRISMA extension for Scoping Reviews (PRISMA ScR), which is an extension of the PRISMA 2020 statement, the most updated version of the Preferred Reporting Items for Systematic Reviews and Meta-Analyses (PRISMA). The PRISMA statement was developed to facilitate transparent and comprehensive reporting of systematic reviews and was updated in 2020 to reflect the most recent developments in the methodology and terminology of systematic reviews [42,43,44]. The PRISMA flow diagram shows the flow of information during the different stages of the present research (Figure 1).

### 2.5. Data Collection and Search Strategy

To form the research question, we also used a simplified form of the PICO (Population, Intervention, Comparison, Outcome) strategy; PCC (Population/Participants, Concept, Context) focuses on evaluating the vegetarian diet as a potential way of preventing and treating cancer patients. In this case, our Population (P) are patients with any kind of cancer, as Concept (C) was considered the adoption of some type of vegetarian diet and Context (C) the protective effects of adopting a vegetarian diet. This strategy enabled us to come up with a resulting question and to perform research based on various bibliometric databases [45].

We queried the three bibliographic databases (Scopus, WoS, and PubMed) in April 2024. The search queries were applied in the title, abstract, and keywords search fields (Table 1). Since the issue being investigated is the association of vegetarian diets with cancer, the first keywords we studied were: “vegetarian”, “vegan”, “plant-based”, and “cancer”, but also their derivatives, such as “vegetarianism”, “veganism”, “lacto-ovo-vegetarian”, “cancers”, “cancerous”, “carcinogenic”, etc. However, both topics (vegetarianism and cancer) studied in this paper are described in the literature in several different terms. Through a search in the Medical Subject Headings (MeSH) thesaurus, other possible keywords came up, which could contribute to the search of these data, such as “tumor”, “neoplasia”, and “malignancy”.

After this carefully planned strategy with multiple searches, each of the above keywords was searched in all three databases to find the most appropriate keyword for each topic to compose a clear and concise search query that identifies as many relevant results as possible. For this purpose, the asterisk (*) wildcard that replaces 0 or more characters was used, whereas the BOOLEAN operators “OR” and “AND” led to the formation of a combined search query.

As shown in Table 1, we performed three distinct search queries in the databases based on our research question because each database has diversifications. Our aim was to include all variations of the keywords associated with cancer and vegetarian diets. In Web of Science we searched in the Topic (TS) field that searches the title, abstract, keyword plus, and author keywords the following query: (((((TS=(tumor*)) OR TS=(neoplas*)) OR TS=(cancer*)) OR TS=(carcin*)) OR TS=(malignan*)) AND (TS=(*vegetarian*) OR TS=(vegan*) OR TS=(plant-based)). In the Scopus database we searched in the Article title, Abstract and Keywords field the query (TITLE-ABS-KEY(cancer*) OR TITLE-ABS-KEY(carcin*) OR TITLE-ABS-KEY(tumor*) OR TITLE-ABS-KEY(malignan*) OR TITLE-ABS-KEY(neoplas*) AND TITLE-ABS-KEY(plant-based) OR TITLE-ABS-KEY(*vegetarian*) OR TITLE-ABS-KEY(vegan*)). Finally, in the PubMed database, we searched in the Title and Abstract fields the query ((cancer*[Title/Abstract]) OR (carcin*[Title/Abstract]) OR (tumor*[Title/Abstract]) OR (malignan*[Title/Abstract]) OR (neoplas*[Title/Abstract])) AND ((vegan*[Title/Abstract]) OR (vegetarian*[Title/Abstract]) OR (plantbased[Title/Abstract])).

The final search queries that were selected returned 7898 publications in total from all three databases (Scopus 3537, Web of Science 2576, PubMed 1785). These results were exported in the appropriate format and then, using the Bibliometrix package of R and the Biblioshiny application were converted into Microsoft Excel files so that they could be merged following a merging method proposed by Caputo and Kargina [39].

### 2.6. Merging Data and Duplicates Removal

After data collection is completed, data are processed. Due to the way they are collected from three different databases, the overlap between the results is inevitable to a certain extent [40]. Therefore, according to the three-step method we followed, we converted Scopus, WOS, and PubMed datasets to bibliography files, then we converted the bib files to Bibliometrix format, and after that, we merged the three datasets manually from Excel. We identified the variables (columns) all three databases have in common, and then we created a master file choosing one of the databases (Web of Science). As shown in Supplementary Table S1, we removed all the variables that pertain to information that is not necessary for the analysis. After that process, 3763 duplicates were excluded leaving 4135 documents left to assess for eligibility.

### 2.7. Eligibility Criteria

We focused mainly on Articles and Reviews written in English language. Thus, 171 publications that were written in another language were excluded (164 of them were in another language, and 6 of them had only an abstract in English). Furthermore, documents that were not Articles or Reviews were also excluded, except for some relevant publications that were published as short surveys, case reports, and reprints; therefore, 519 scientific publications that did not fit these parameters were not included in the sample. Finally, 18 publications with a final publication date of 2024 were removed from the sample as we studied all the articles and reviews regarding our research question published until 2023. In total, we used 3427 scientific publications (articles, reviews, short surveys, case reports, and reprints) for this bibliometric analysis.

## 3. Results

In the results section, the main findings from the bibliometric analysis of these data will be presented, including keywords, thematic evolution, connections and collaborations among countries, authors, and scientific institutions, as well as information on the annual production of scientific articles, primary publishing sources, and their respective countries of origin.

### 3.1. Main Information

Table 2 presents the main information of the final bibliometric collection that emerged and was used in the present study. The total number of scientific documents examined was 3427, the majority of which consisted of scientific articles (article: 2364) and reviews (review: 1050). Additionally, there were some short surveys (short survey: 10), a case report (case reports: 1), a reprint (reprint: 1), and an uncategorized document included as well. These data have been published from 1930 to 2023 and originate from a total of 1407 sources. A total of 14,005 authors were involved in their production, selecting 7645 keywords. A total of 286 documents are single-authored, while their respective authors include 243 individuals. For articles with more than one author, the average number of authors per document is 5.826. The degree of international co-authorship reached 19.84%.

### 3.2. Annual Scientific Production

**Figure 2:**
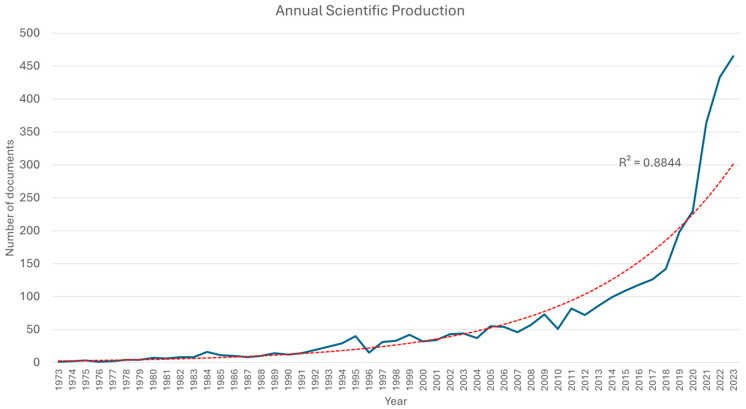
Annual scientific production of documents containing the selected search terms for this study since 1973. The red dashed line represents the exponential trend line with an R^2^ value of 0.8844.

**Figure 3:**
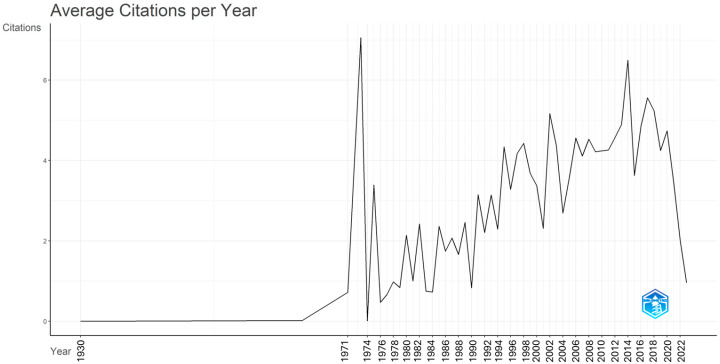
Average annual citation of the scientific documents in the bibliometric collection under study using Bibliometrix R-package.

Notably, in the annual production of scientific articles containing the selected terms for this study, there is one article in 1930, another in 1964, and a subsequent one in 1971, while the systematic production of scientific articles on an annual basis begins from 1973 onwards. A significant increase in annual production is observed in 1984, with 16 articles, followed by a rising trend from 1994 onwards. The peak in production is noted in 2023, with 465 documents. Figure 2 illustrates the annual scientific production of scientific documents since 1973, which fits an exponential trend line with an R^2^ value of 0.8844. Furthermore, Figure 3 displays the average annual citation count of the articles (average citations per year).

### 3.3. Sources

Data for this study originate from a total of 1407 sources. Table 3 presents the ten most significant sources, which are the “*American Journal of Clinical Nutrition*” with 131 publications, “*Nutrients*” with 123 publications, “*Molecules*” with 42 publications, the “*British Journal of Nutrition*” with 36 publications, the “*Journal of Nutrition*” with 35 publications, the “*European Journal of Nutrition*”, the “*International Journal of Molecular Sciences*” and “*Nutrition and Cancer—An International Journal*” with 29 publications each, and both the “*Frontiers in Nutrition*” and “*PLOS One*” with 28 publications each. From these top 10 journals, 14.88% of all data are derived.

**Figure 4:**
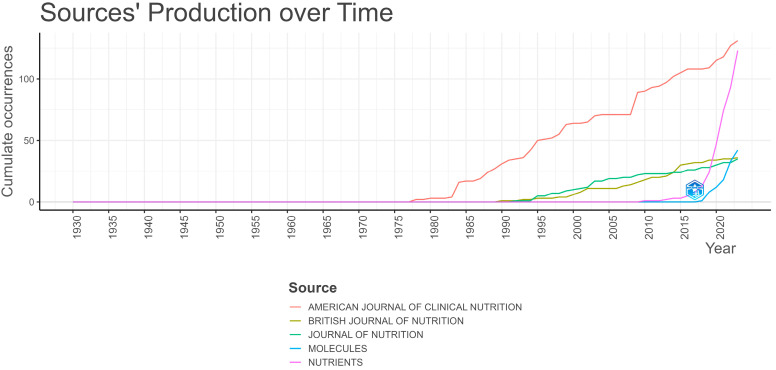
Evolution of document production from the most significant journals—sources over time using Bibliometrix R-package.

**Figure 5:**
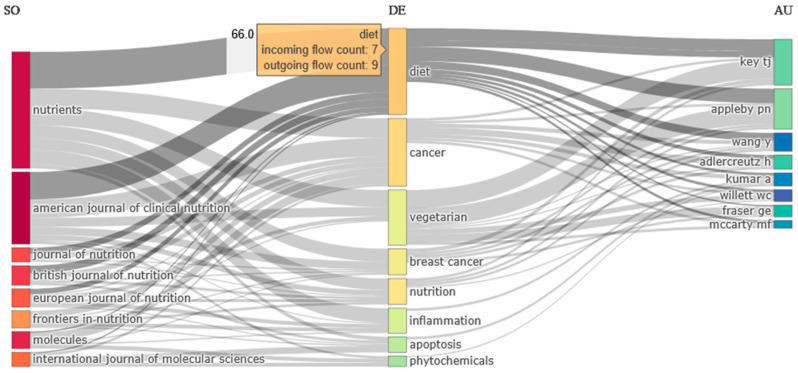
Interrelation of Sources, Keywords, and Authors in the bibliometric collection under study, showing as an example of the incoming and the outgoing flow of the keyword diet.

According to Bradford’s law, these top 10 journals, along with 45 others, constitute the core sources. Although constituting only 3.91% of the total, these 55 scientific journals account for 33.21% of all data (1138 articles) in the study collection. Figure 4 illustrates the temporal evolution of article production from the top five journals. The “*American Journal of Clinical Nutrition*” began addressing the studied terms early and with considerable intensity, followed by the “*British Journal of Nutrition*” and the “*Journal of Nutrition*”. Conversely, articles related to the search terms began to appear in “*Molecules*” and “*Nutrients*” only in the last 5 and 13 years, respectively, to such an extent that their contribution is crucial. Figure 5 presents the interrelation among the top sources, keywords, and authors of the specific bibliometric collection. Notably, most articles containing the keywords “diet” and “cancer” originate from the top scientific journals of the dataset. Furthermore, the significant contributions of authors Paul N. Appleby and Timothy J. Key, who have extensively explored vegetarianism and its health implications, are noteworthy [6,46,47,48,49].

### 3.4. Authors

**Figure 6:**
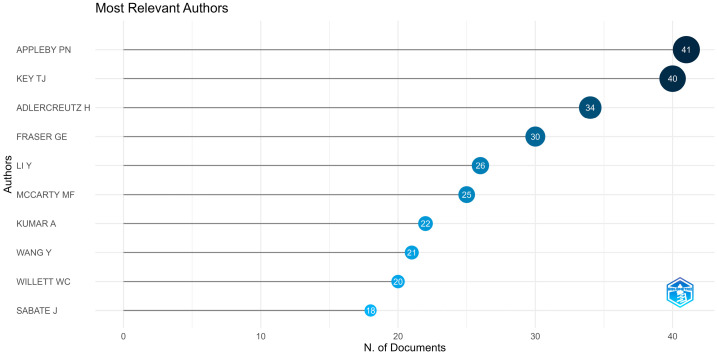
The top ten authors in terms of data production within the bibliometric collection under study using Bibliometrix R-package.

**Figure 7:**
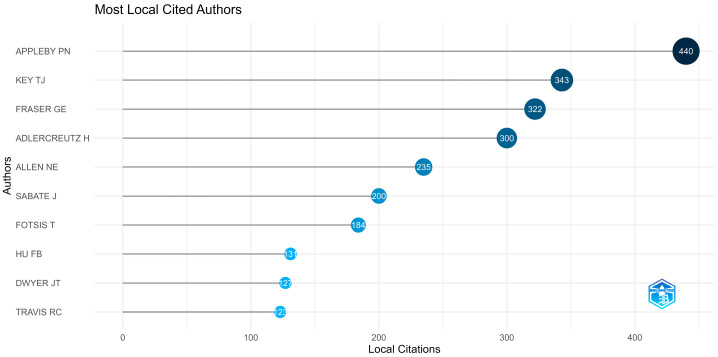
The ten most locally cited authors in the bibliometric collection under study using Bibliometrix R-package.

**Figure 8:**
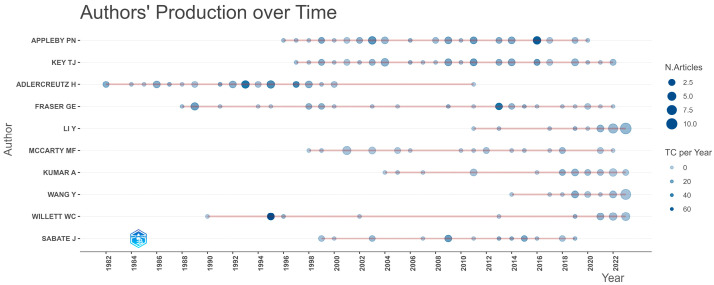
Author’s document production over time using Bibliometrix R-package.

The contribution of Appleby and Key becomes more apparent from the analysis presented in Figure 6 and Figure 7, indicating that they are the authors with the highest article production in this collection and are also the most frequently cited across all the scientific articles under study (most locally cited authors). Figure 8 represents the authors’ document production over time.

### 3.5. Collaboration

**Figure 9:**
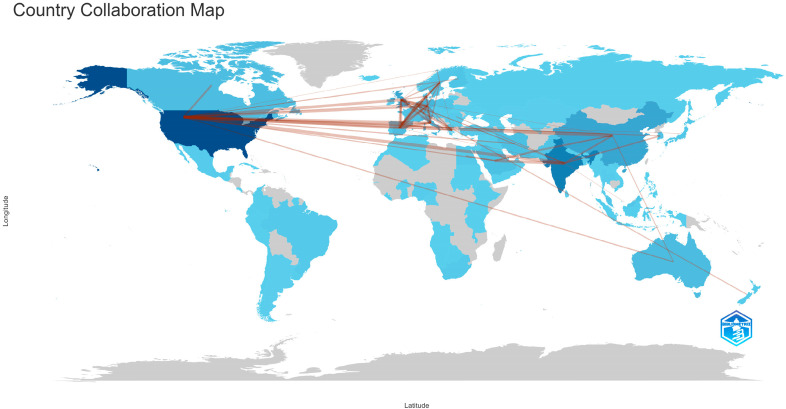
World map depicting the production of scientific files by country. The intensity of color corresponds to the number of publications. Red lines indicate collaborations between countries using Bibliometrix R-package.

The publications comprising our study’s bibliometric collection originate from 110 countries. Figure 9 illustrates the countries from which these scientific data in this study emanate, with darker shades of blue indicating an increase in the number of published data from each respective country. The country of publication for each scientific file is determined by the affiliation of the authors. The countries with the most prominent scientific output are the United States of America (2792 scientific publications), India (1501 scientific publications), China (995 scientific publications), the United Kingdom (674 scientific publications), and Italy (555 scientific publications). The same figure also depicts scientific collaborations between countries, represented by red lines. The countries with the most connections are the United States of America with China (50 collaborations), the United States of America with India (43 collaborations), India with Saudi Arabia (34 collaborations), the United States of America with the United Kingdom (34 collaborations), the United Kingdom with Germany (26 collaborations), the United Kingdom with Italy (26 collaborations), the United States of America with Germany (26 collaborations), and the United States of America with Italy (26 collaborations).

### 3.6. Keywords and Trend Topics

**Figure 10:**
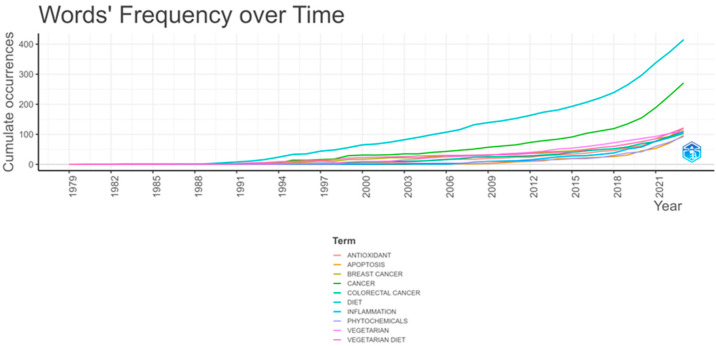
The temporal evolution of the top ten most frequent keywords chosen by authors using Bibliometrix R-package.

Upon analyzing the keywords chosen by authors for their works, it becomes evident that the words “diet”, “cancer”, and “vegetarian diet” emerge as the most prevalent, aligning with the thematic focus of the study (Table 4). Tracking the evolution of the most common keywords used by scientists reveals that the term “vegetarian diet” is chronologically first, dating back to 1980, followed by the terms “vegetarian” (1987) and “diet” (1988). Although the term “cancer” emerged later (1992), it was chosen with significantly increased frequency (Figure 10).

**Figure 11:**
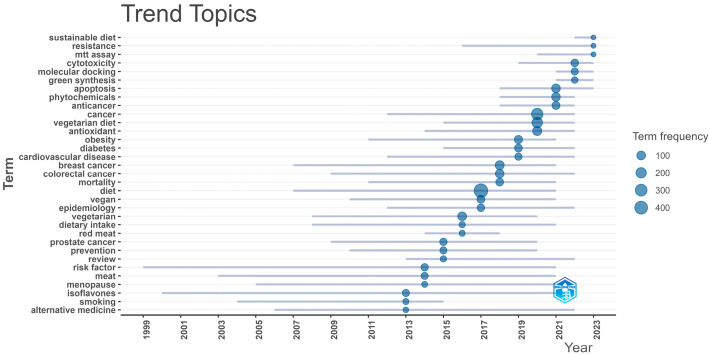
Trending Topics during the Period 2013–2023 reflecting the evolution of authors’ keywords using Bibliometrix R-package.

Through the evolution of trending topics indicated by keywords chosen by authors, it is observed that vegetarianism (“vegetarian”) began to be studied more intensively from 2008, while terms related to veganism (“vegan”) and “cancer” emerged slightly later, around 2010 and 2011 respectively. The antioxidant properties of vegetarianism are immediately addressed thereafter, while the trend of phytochemicals emerged in 2017. On the contrary, studies on “isoflavones” have been documented as early as 2000. From 2020 onwards, the trending topic of ‘MTT assay’ emerges. The MTT assay is a widely used colorimetric method for assessing cell metabolic activity, commonly employed to evaluate cell viability, proliferation, and cytotoxicity, as well as the efficacy of drugs and anticancer agents [50,51]. This signifies the scientific progress of contemporary cancer research. In recent years, in addition to health impacts, the sustainability of this dietary approach (“sustainable diet”) has also started to be investigated, as depicted in Figure 11.

### 3.7. Thematic Evolution

**Figure 12:**
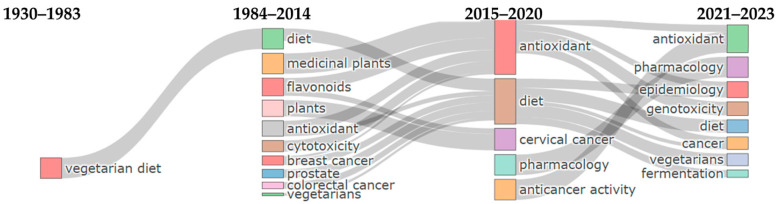
Thematic evolution based on authors’ keywords across four different time periods: 1930–1983, 1984–2014, 2015–2020, and 2021–2023.

Diving into the evolution of thematic trends in cancer and vegetarianism-related research, a chronological analysis of author-selected keywords was conducted (Figure 12). The studied time periods were selected based on scientific output. Hence, the first period (1930–1983) reflects a single-digit or zero annual publication count on the subject. During this time frame, the few published works mainly focused on the vegetarian diet. In the second period (1984–2014), the annual production of relevant literature studies remained stable and systematic, expanding the thematic scope. Concerning vegetarianism, the focus widened to encompass diet in general, while topics such as medicinal plants, plants, flavonoids, antioxidants, cytotoxicity, and different types of cancer emerged, predominantly targeting cancer-related aspects. In the subsequent period (2015–2020), there was a further increase in annual scientific output, reaching triple digits. Two entirely new topics emerged during this period: pharmacology and anticancer activity. Most of the literature on plants appeared as a new theme targeting cervical cancer, along with a small fraction of flavonoids. All other topics that had emerged in the previous period formed two new thematic umbrellas that already existed: diet and antioxidants. The final period studied focused on the themes of the preceding three years (2021–2023), witnessing an exponential increase in scientific output on the subject. Antioxidants, pharmacology, and diet remained the core themes during this period. The new concepts that emerged primarily branch off from diet and antioxidant themes. Hence, the new terms for the core topics of the most recent chronological period include fermentation, epidemiology, cancer, vegetarians, and genotoxicity.

### 3.8. Co-Occurrence Network

**Figure 13:**
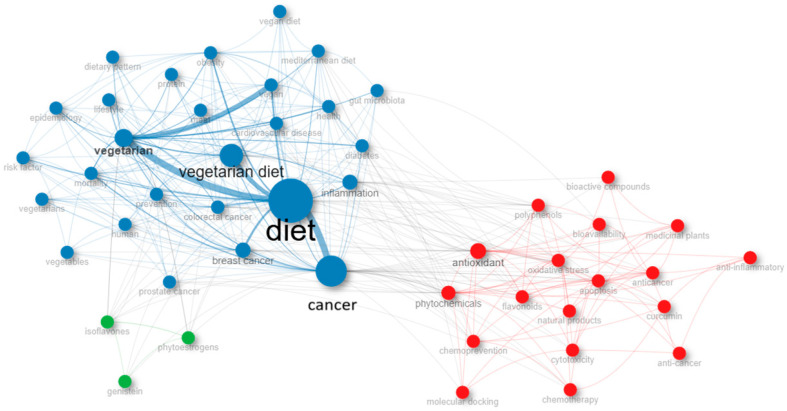
Co-occurrence network of authors’ keywords in the bibliometric collection under study.

As depicted in Figure 13, 48 of the keywords utilized by the authors within this specific bibliometric collection co-occur and are categorized into three distinct groups. The predominant group is highlighted in blue and focuses on the correlation between diet and cancer from an epidemiological and lifestyle perspective (e.g., diet, cancer, vegetarian diet, health, vegan, Mediterranean diet, epidemiology, lifestyle, risk factor, gut microbiota, diabetes, obesity, etc.). The keywords of the next group are centered around biochemistry (e.g., antioxidant, phytochemicals, bioactive compounds, apoptosis, bioavailability, etc.) and are displayed in red. The final group, represented in green, consists of just three terms (genistein, phytoestrogens, and isoflavones), which essentially represent bioactive constituents found in plant-based foods.

### 3.9. Most Local Cited Documents

The most locally cited documents in a bibliometric collection are those that have received the highest number of citations from other works in the same dataset. These documents serve as key reference points while indicating significant contributions to the research field. The frequent citations suggest that these works contain crucial findings, methodologies, or theoretical frameworks that are foundational to subsequent studies. Examining these influential documents reveals the foundational studies that have propelled advancements in the field. The most locally cited documents in the bibliometric collection of this study are shown in Table 5 and are further discussed in the discussion section.

## 4. Discussion

### Summary of Evidence

Based on data collected for this study, the initial link between a vegetarian diet and cancer was established in 1930 by Parkes in a paper about diverticulitis. Parkes noted that diverticulitis sometimes occurs as a consequence of carcinomas and suggested that a lacto-vegetarian diet could be part of the treatment [59]. This connection remained unexplored in the literature for over 30 years until 1964, when Bazikian published a study on the spread of malignant tumors in Armenia [60]. In 1971, Aries et al. conducted a study comparing the microbial flora and steroid concentrations in the feces of vegetarians and non-vegetarians to investigate the link between meat consumption and gastrointestinal cancer. This study revealed a slight protective effect of vegetarianism against the production of potentially carcinogenic bile acids [61]. From 1973 onwards, there has been a consistent increase in scientific publications exploring the relationship between vegetarianism and cancer.

The core scientific sources of bibliometric data collection analyzed in this study primarily focus on nutrition and molecular sciences, reinforcing the fundamental connection between diet and health. The terms “diet”, “cancer”, and “vegetarian diet” are the most frequently encountered keywords, which aligns with this study’s focus. Regarding authorship, Appleby and Key are the most prolific contributors to this topic, with 41 publications in this collection for Appleby and 40 publications for Key. They also have the highest number of local citations (440 citations for Appleby and 343 citations for Key).

By examining the most locally cited papers in our bibliometric analysis, the top position is occupied by the work of Davey et al., which details the sociodemographic and lifestyle characteristics and the nutrient intake of participants in the EPIC-Oxford study. The high citation frequency of this article underscores its importance in the scientific community studying cancer and vegetarianism. The EPIC-Oxford study is one of the largest cohort studies on a vegetarian population, aiming to link dietary patterns with various forms of cancer [52]. Following this is the research by Satija et al., which focuses on the effects of plant-based food consumption on cardiovascular diseases. This study introduces the concept of vegetarian diet quality, emphasizing that merely excluding animal products does not necessarily constitute a healthy diet, thus adding an important consideration for future research and dietary guidelines [53].

The results of a large cohort study support the beneficial effects of a vegetarian diet against certain types of cancer. The dietary habits and medical history of a large population of Seventh-day Adventists were studied over an 11-year period. This group was chosen to minimize the impact of confounding factors such as smoking and alcohol consumption. This study concluded that increased consumption of fruits, vegetables (especially tomatoes), and legumes in a vegetarian diet has a statistically significant positive impact on the risk of prostate, colorectal, and lung cancer [54]. Between 2002 and 2007, the Adventist Health Study 2 (AHS-2) was conducted on a similar population. The AHS-2 concluded that individuals following any model of a vegetarian diet exhibited statistically significant lower mortality rates compared with non-vegetarians, although there was no difference in cancer-related mortality. Early analyses of this study identified a lower risk of gastrointestinal cancers and cancers of the breast and cervix among vegetarians [56].

On the other hand, the European EPIC-Oxford study did not find a statistical difference between vegetarians and the general population regarding colorectal cancer. However, it showed that vegetarians have a 62% lower risk of developing stomach cancer compared with meat-eaters, possibly because of the absence of processed meat consumption, such as deli meats [24,49]. Additionally, the same study concluded that vegetarians and vegans have 10% and 18% lower risks, respectively, of developing any type of cancer compared with meat consumers. Moreover, according to the EPIC-Oxford study, vegetarians have a 63% lower risk of developing hematological cancers compared with non-vegetarians. Conversely, they have a 90% higher risk of cervical cancer compared with those who consume animal products. This last finding is unexpected but of marginal statistical significance [24].

Regarding mortality rates, the results of the same study did not reveal any statistically significant differences between vegetarians and non-vegetarians, although vegetarians exhibited a 19% lower risk of death from ischemic heart disease compared with meat-eaters. Vegetarians tend to have lower body weight and avoid smoking, habits that contribute positively to overall health [62]. The results from the AHS-2 study do not fully align with the aforementioned findings, as they indicate significantly lower mortality and a reduced risk of developing colon cancer. The differing lifestyle and dietary habits between the two populations are the primary explanations for these discrepancies, as the sample in the British study largely comprises individuals who are health conscious [55]. In a similar context, a meta-analysis by Huang et al. concluded that vegetarians show 29% lower ischemic heart disease mortality and 18% lower incidence of cancer compared with non-vegetarians. Additionally, the overall mortality for vegetarians was found to be 9% lower, with a 16% lower mortality from circulatory diseases and a 12% lower mortality from cerebrovascular diseases compared with non-vegetarians [20]. These findings are highly significant, as evidenced by the fact that these papers are among the most frequently cited in the current bibliometric collection.

Based on the findings from the AHS-2 and EPIC-Oxford studies, Craig decided to investigate the potential health impacts of veganism. His research concluded that vegans tend to have a lower BMI, a reduced risk of cardiovascular disease, and lower levels of cholesterol and blood pressure. However, since vegans often have lower intakes of calcium and vitamin D compared with individuals following other dietary patterns, there is an increased risk of fractures and low bone density. Regarding cancer, the findings are not yet fully clarified. It is recommended that vegans ensure adequate intake of nutrients such as vitamins B12 and D, calcium, omega-3 fatty acids (especially DHA), and zinc to prevent potential deficiencies and associated health problems [21].

The 1982 study by Goldin et al. is one of the most frequently cited studies, as it provided new insights into the relationship between dietary habits and cancer risk. This study found that women following a vegetarian diet have increased fiber intake, leading to greater bowel motility. This increased bowel activity results in higher fecal production and the excretion of estrogens through feces [57]. Since estrogens are associated with the development of breast cancer, their excretion through increased bowel motility may act as a protective factor against this type of cancer [63].

The results of the previous studies, highlighting the beneficial effects of vegetarianism on health, the importance of a balanced diet emphasizing specific nutritional components, and the environmental benefits, are also confirmed by the highly cited article outlining the position of the Academy of Nutrition and Dietetics on vegetarian diets [58]. Additionally, according to the findings of the meta-analysis of Huang et al. (2012), it suggests that people who follow a vegetarian diet have significantly lower ischemic heart disease mortality and are less likely to develop cancer overall than non-vegetarians. [20].

The present study is, to our knowledge, the first to attempt to examine and evaluate the relationship and impact of a vegetarian diet on cancer through bibliometric analysis. The significance of its novel findings is reinforced by adherence to the proper and recommended workflow of scientific mapping as suggested for the bibliometric process [33]. One of the key advantages of this study is that the final bibliometric collection used in the analyses combines findings from three different databases (Scopus, PubMed, and Web of Science). This significantly broadens the scope of the sample under study and reduces the chances of omitting important literature studies related to the topic, thereby strengthening the contribution of the findings.

During the process of merging data from the databases and removing duplicate entries, both automated and manual methods were employed to find and delete unnecessary data. Although manual processes can sometimes be a source of errors, in this case, they are considered beneficial as they ensure comprehensive control over the results and minimize the likelihood of failing to eliminate duplicate entries from the sample. Additionally, the manual method was crucial for familiarizing with the entire dataset and identifying extraction problems, such as records with incomplete data, which might arise from solely relying on automated methods for merging and deletion [39].

However, this bibliometric review is not without its limitations, some of which stem from the bibliometric analysis process itself. Since bibliometric analyses largely rely on the number of citations and references, their results should be interpreted with caution. This also applies to the findings of this study. In the scientific community, it is commonly assumed that the number of citations and references is an indicator of the importance and impact of research. This is a misconception, as the citation system has its own advantages and disadvantages. The main disadvantage is that the citation system cannot assess the quality of the cited studies [64,65]. As our analyses heavily rely on citations of scientific papers, they cannot evaluate the quality of the studies being reviewed. Additional factors that prevent the evaluation of the quality and reliability of the studies in our sample include the volume and heterogeneity of the sample.

Furthermore, by using PCC instead of the PICO strategy, our research question remained broader as the main focus of it, as a scoping review using bibliometric analysis, was to map the literature regarding vegetarianism and cancer rather than to gather studies with certain characteristics in such multidimensional topics. PICO is broadly accepted as a strategy to create research questions and successfully plan research; however, it may be more effective for other types of studies (such as interventional studies) than scoping studies as the present study [66]. Further research in evidence-based research to measure the effectiveness of vegetarian diets in certain cancer types in specific populations could employ the PICO strategy in order to establish valuable insights.

This study did not impose any temporal limitations on the publication dates of the papers included in the final sample. This approach was taken to include all possible data related to the topic under study. If our study had been limited to the literature of recent years, where scientific production is more intensive, and there is significant advancement in health sciences, the results might have been different. Nonetheless, scientific research on this topic is undoubtedly rapidly evolving, with the ultimate goal of elucidating the relationship between vegetarianism and cancer. The quality of a vegetarian diet is a crucial issue that should be the focus of future research. Although more recent publications were not included in our quantitative analysis, scientists perform studies examining the possibility of the positive effects of plant-based diets in cancer prevention and treatment [12,13,25,26,27,28] fact that encourages further research on the topic.

## 5. Conclusions

The present study is the first to examine the relationship between vegetarianism and cancer through the lens of bibliometrics, addressing questions regarding the annual increase in the production of related articles, their sources, thematic fields, and geographical distribution. Additionally, the analyses conducted generated a co-occurrence network for the reliable identification of authors’ key terms and their relationships, investigated trending topics and their evolution over time, and reviewed articles with the most significant impact on the scientific community based on the analysis results.

Our findings demonstrate a consistently increasing number of scientific articles examining the correlation between vegetarian diets and the incidence or progression of cancer. However, due to the complexity of the topic, their results are often contradictory. A significant number of studies suggest a protective effect from adopting a vegetarian dietary model against certain types of cancer, while other studies have not been able to confirm these findings. Studies focusing on the impact of vegetarianism on cancer while also considering the quality of vegetarian diets, are essential to obtain a clear answer to this question.

## Figures and Tables

**Table 1:** Topics studied in the present paper and the search terms that lead to the final search query for each database.

Topic	Search Terms	Keyword	Databases Final Search Query		
			WoS	Scopus	PubMed
Vegetarian Diet	vegan vegans veganism	vegan*	(((((TS=(tumor*)) OR TS=(neoplas*)) OR TS=(cancer*)) OR TS=(carcin*)) OR TS=(malignan*)) AND (TS=(*vegetarian*) OR TS=(vegan*) OR TS=(plant-based))	(TITLE-ABS-KEY(cancer*) OR TITLE-ABS-KEY(carcin*) OR TITLE-ABS-KEY(tumor*) OR TITLE-ABS-KEY(malignan*) OR TITLE-ABS-KEY(neoplas*) AND TITLE-ABS-KEY(plant-based) OR TITLE-ABS-KEY(*vegetarian*) OR TITLE-ABS-KEY(vegan*))	((cancer*[Title/Abstract]) OR (carcin*[Title/Abstract]) OR (tumor*[Title/Abstract]) OR (malignan*[Title/Abstract]) OR(neoplas*[Title/Abstract])) AND ((vegan*[Title/Abstract]) OR(vegetarian*[Title/Abstract]) OR (plantbased[Title/Abstract]))
	vegetarian vegetarians lacto-ovovegetarian vegetarianism	vegetarian* (PubMed) *vegetarian* (WoS, Scopus)			
	plant-based plant-based	plant-based			
Cancer	tumor tumors tumorigenesis tumoral	tumor*			
	neoplasia neoplasm neoplasms	neoplas*			
	cancer cancers cancerous cancerization	cancer*			
	carcinoma carcinogenic carcinogens	carcin*			
	malignant malignancy malignancy	malignan*			

* symbol is part of the search queries.

**Table 2:** Main information of the final bibliometric collection.

Description	Results
MAIN INFORMATION ABOUT DATA	
Timespan	1930–2023
Sources (Journals, Books, etc.)	1407
Documents	3427
Annual Growth Rate %	6.83
Document Average Age	9.86
Average citations per doc	39.34
References	188,267
DOCUMENT CONTENTS	
Keywords Plus (ID)	16,849
Author’s Keywords (DE)	7645
AUTHORS	
Authors	14,005
Authors of single-authored docs	243
AUTHORS COLLABORATION	
Single-authored docs	286
Co-Authors per Doc	5.26
International co-authorships %	19.84
DOCUMENT TYPES	
article	2364
case reports	1
reprint	1
review	1050
short survey	10
case reports	1

**Table 3:** Most relevant sources by the number of published documents.

Sources	Articles
American Journal of Clinical Nutrition	131
Nutrients	123
Molecules	42
British Journal of Nutrition	36
Journal of Nutrition	35
European Journal of Nutrition	29
International Journal of Molecular Sciences	29
Nutrition And Cancer-an International Journal	29
Frontiers in Nutrition	28
PLOS One	28

**Table 4:** The ten most frequently used keywords by the authors.

Words	Occurrences
diet	474
cancer	271
vegetarian diet	205
antioxidant	120
breast cancer	112
vegetarian	111
inflammation	108
colorectal cancer	97
apoptosis	96
phytochemicals	93

**Table 5:** The ten most locally cited documents in the bibliometric collection of this study.

Document	Year	Local Citations
Davey G.K., 2003, Public Health Nutr [52]	2003	73
Satija A., 2017, J Am Coll Cardiol [53]	2017	67
Fraser G.E., 1999, Am J Clin Nutr [54]	1999	62
Key T.J., 2009, Am J Clin Nutr-A [48]	2009	58
Fraser G.E., 2009, Am J Clin Nutr [55]	2009	50
Orlich M.J., 2013, Jama Intern Med [56]	2013	50
Craig W.J., 2009, Am J Clin Nutr [21]	2009	47
Huang T., 2012, Ann Nutr Metab [20]	2012	47
Goldin B.R., 1982, New Engl J Med [57]	1982	45
Melina V., 2016, J Acad Nutr Diet [58]	2016	41

## Data Availability

Data that support the findings of this study are available from the corresponding author upon reasonable request.

## Supplementary Materials

The following supporting information can be downloaded at: https://www.mdpi.com/article/10.3390/epidemiologia6020023/s1, Table S1: Variables—columns common to the three databases (Web of Science, Scopus, and PubMed).
