# Supplementary material for: Studying the Role of Vegetarianism as a Potential Strategy for Cancer Prevention and Treatment, a Bibliometric Analysis
Source: Epidemiologia (Basel). 2025 May 5;6(2):23. doi: 10.3390/epidemiologia6020023 (PMC12101372; doi:10.3390/epidemiologia6020023)
Supplement: Supplementary file 1 [file epidemiologia-06-00023-s001.zip › epidemiologia-3513797-supplementary.pdf]

# Supplementary Material

This appendix is a part of the original submission.

**Table S1.** Variables – columns common to the three databases (Web of Science, Scopus and Pubmed).

| Variable | Explanation | Web of Science | Scopus | Pubmed |
|---|---|---|---|---|
| AU | Authors | x | x | x |
| DE | Author Keywords | x | x | x |
| ID | Keywords Plus | x | x | x |
| C1 | Author Address | x | x | x |
| CR | Cited References | x | x | x |
| AB | Abstract | x | x | x |
| DI | Digital Object Identifier (DOI) | x | x | x |
| SN | International Standard Serial Number (ISSN) | x | x | x |
| SO | Publication Name | x | x | x |
| LA | Language | x | x | x |
| PP | Pages | x | x | x |
| TC | Web of Science Core Collection Times Cited Count | x | x | x |
| TI | Document Title | x | x | x |
| DT | Document Type | x | x | x |
| VL | Volume | x | x | x |
| PY | Year Published | x | x | x |
| RP | Reprint Address | x | x | x |
| DB | Database | x | x | x |
| J9 | 29-Character Source Abbreviation | x | x | x |
| AU_UN | University of affiliation for each co-author | x | x | x |
| AU1_UN | University of affiliation for the corresponding author | x | x | x |
| AU_UN_NR | Not Recognized Affiliations | x | x | x |
| SR_FULL | Short Full-Reference | x | x | x |
| SR | Short Reference | x | x | x |
| PA | Publisher Address | x | x | |
| AR | Article Number | x | x | |
| BE | Editors | x | x | |
| FU | Funding Agency and Grant Number | x | x | |
| FX | Funding Text | x | x | |
| BN | International Standard Book Number (ISBN) | x | x | |
| JI | ISO Source Abbreviation | x | x | |
| PN | Part Number | x | x | |
| PU | Publisher | x | x | |
| affiliations | affiliations | x | | |
| EM | E-mail Address | x | | |
| BO | Book Title | x | | |
| Da | Date this report was generated | x | | |
| GA | Document Delivery Number | x | | |
| eissn | Electronic International Standard Serial Number | x | | |
| earlyaccessdate | Early Access date | x | | |
| meeting | Meeting | x | | |
| month | Month | x | | |
| note | Note | x | | |
| NR | Cited Reference Count | x | | |

| Variable | Explanation | Web of Science | Scopus | Pubmed |
|---|---|---|---|---|
| Oa | Open Access Indicator | x | | |
| orcid.numbers | ORCID numbers | x | | |
| organization | Organization | x | | |
| SC | Research Areas | x | | |
| researcerid.numbers | Research ID numbers | x | | |
| SE | Book Series Title | x | | |
| UT | Accession Number | x | | |
| usage.count.last.180.days | Usage Count (Last 180 Days) | x | | |
| U2 | Usage Count (Since 2013) | x | | |
| web.of.science.categories. | Web Of Science Categories | x | | |
| web.of.science.index | Web of Science Index | x | | |
| chemicals_cas | Chemical CAS | | x | |
| coden | CODEN | | x | |
| manufacturers | manufacturers | | x | |
| molecular_seqnumbers | Mole | | x | |
| page_count | Page count | | x | |
| PM | PMC Identifier | | x | |
| sponsors | Sponsors | | x | |
| tradenames | Tradenames | | x | |
| url | URL | | x | |
| AF | Affiliation | | | x |
| AID | Article Identifier | | | x |
| OT | Other Term | | | x |
| PHST | Publication History Status Date | | | x |
| OI | Other ID | | | x |
| BTI | Book Title | | | x |
| CI | Copyright Information | | | x |
| CIN | Comment In | | | x |
| CN | Corporate Author | | | x |
| COIS | Conflict of Interest statement | | | x |
| CON | Comment On | | | x |
| CRDT | Create Date | | | x |
| CTDT | Contribution Date | | | x |
| DCOM | Completion Date | | | x |
| DEP | Date of Electronic Publication | | | x |
| ECI | Expression of Concern In | | | x |
| EDAT | Entry Date | | | x |
| EFR | Erratum For | | | x |
| EIN | Erratum In | | | x |
| FIR | Full Investigator Name | | | x |
| FPS | Full Personal Name as Subject | | | x |
| GN | General Note | | | x |
| GR | Grants and Funding | | | x |
| IS | ISSN | | | x |
| IR | Investigator | | | x |
| IRAD | Investigator Affiliation | | | x |
| JID | NLM Unique ID | | | x |
| LID | Location ID | | | x |
| LR | Modification Date | | | x |
| MHDA | MeSH Date | | | x |
| MID | Manuscript Identifier | | | x |
| OAB | Other Abstract | | | x |
| OABL | Other Abstract Language | | | x |
| OID | Other ID | | | x |
| OTO | Other Term Owner | | | x |
| OWN | Owner | | | x |
| PB | Publisher | | | x |

| Variable | Explanation | Web of Science | Scopus | Pubmed |
|---|---|---|---|---|
| PL | Place of Publication | | | **x** |
| PMC | PubMed Central Identifier | | | **x** |
| PMCR | PMC Release | | | **x** |
| PMID | PubMed Unique Identifier | | | **x** |
| PS | Personal Name as Subject | | | **x** |
| PST | Publication Status | | | **x** |
| RF | Number of References | | | **x** |
| RIN | Retraction In | | | **x** |
| RN | EC/RN Number | | | **x** |
| SB | Subset | | | **x** |
| SI | Secondary Source ID | | | **x** |
| SPIN | Summary For Patients In | | | **x** |
| STAT | Status Tag | | | **x** |
| TT | Transliterated Title | | | **x** |
